# Supplementary material for: Epiregulin as an Alternative Ligand for Leptin Receptor Alleviates Glucose Intolerance without Change in Obesity
Source: Cells. 2022 Jan 26;11(3):425. doi: 10.3390/cells11030425 (PMC8834548; doi:10.3390/cells11030425)
Supplement: Supplementary file 1 [file cells-11-00425-s001.zip › cells-1541961-supplementary.pdf]

Supplemental data.

Content: nine supplemental figures and figure legends.

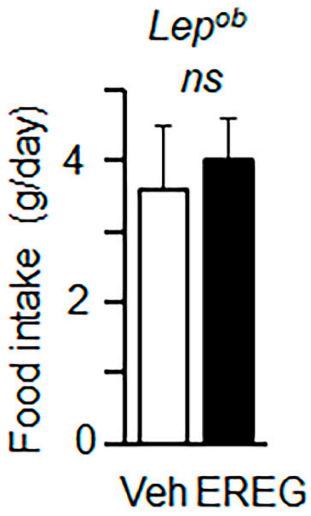

Supplemental Figure S1. The lack of EREG effect on food intake in *Lep*^{ob} mice.

Average daily food uptake of *Lep*^{ob} male mice treated with vehicle (white bar) or EREG (black bar, 50 ng/g BW) as described in Figure 1, A-F (n = 7/group).

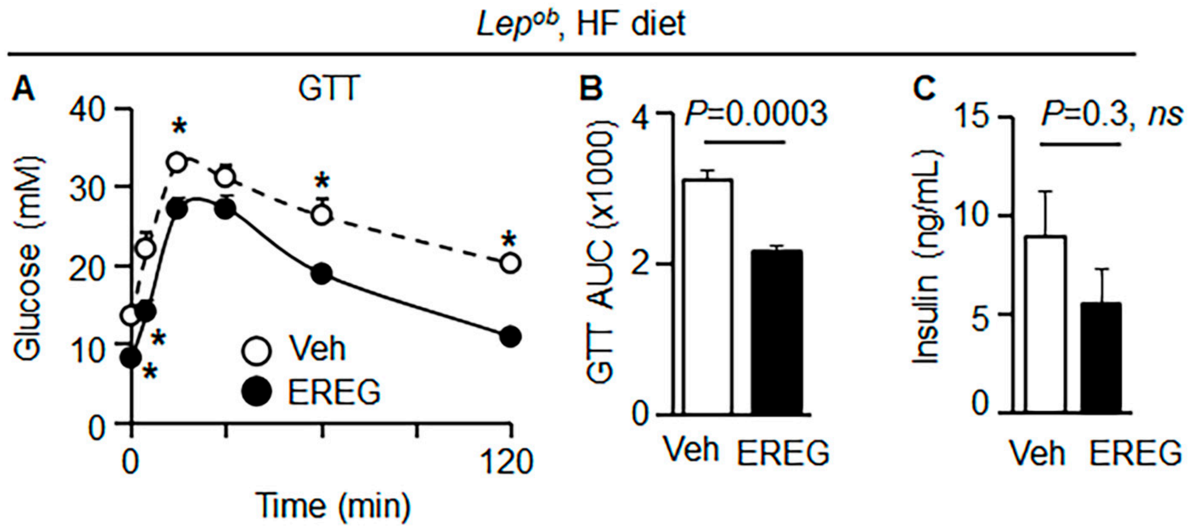

Supplemental Figure S2. EREG improved glucose intolerance in *Lep^{ob}* mice on a high-fat diet.

Six-week-old *Lep^{ob}* male mice (B6.V-Lepob/J strain containing spontaneous mutation in the gene encoding leptin congenic on C57BL/6J) were purchased from the Jackson Laboratory (stock number 000632, n = 10). *Lep^{ob}* mice were fed with a high-fat diet (D12451, Research Diet Inc) for 30 days. The metabolic characterization of these mice was described {Yasmeen, 2018 #862}. Then, mice were randomly assigned into two groups:

(1) Control *Lep^{ob}* mice group, injected with 0.1mL sterile PBS (n = 5), and

(2) EREG treated group of *Lep^{ob}* mice (n = 5), injected intraperitoneally with PBS containing EREG (2.7 ng/g body weight, that corresponds to 60 ng/epididymal fat depot). EREG was injected every other day for 6 weeks.

(**A**) The glucose levels in the blood of the control and EREG treated *Lep^{ob}* mice during the GTT test pair-fed a high-fat diet. Asterisks,-significant differences between control and EREG-treated groups of *Lep^{ob}* mice (*t*-test). (**B**) Area under the curve was measured based on GTT kinetics. The *t*-test was used for statistical comparison. (**C**) Insulin levels in plasma in the control and EREG-treated *Lep^{ob}* mice.

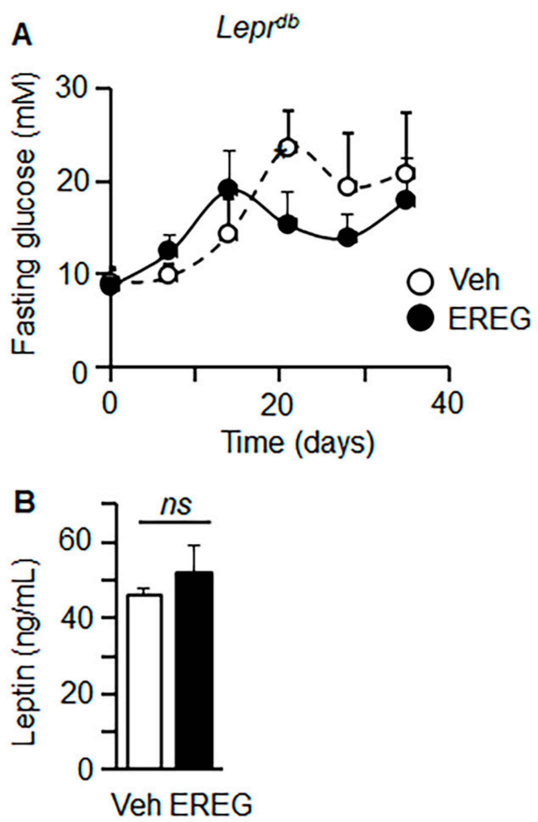

Supplemental Figure S3. EREG did not affect fasting glucose and leptin levels in plasma in *Lepr^{db}* mice

(**A**) Fasting glucose kinetics in *Lepr^{db}* mice treated with vehicle (open circles) or EREG (50ng/g BW, closed circles) as described in Fig.1 G-L (n = 6/group). (**B**) The levels of leptin levels in plasma in the same *Lepr^{db}* mice. Leptin concentrations were measured by ELISA. Student's *t*-test. *ns*, not significant ($p > 0.05$).

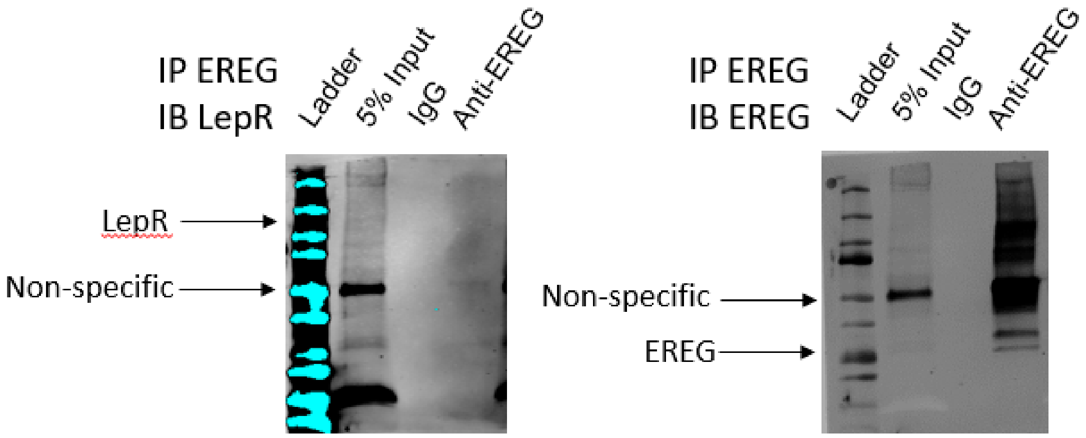

Supplemental Figure S4. Immunoprecipitation with human anti-EREG antibody in adipose tissue isolated from an obese insulin-resistant patient.

Low expression of LepR in obese insulin-resistant patients was observed (left panel), in agreement with documented previous reports [26]. Right panel shows the EREG expression in these tissues.

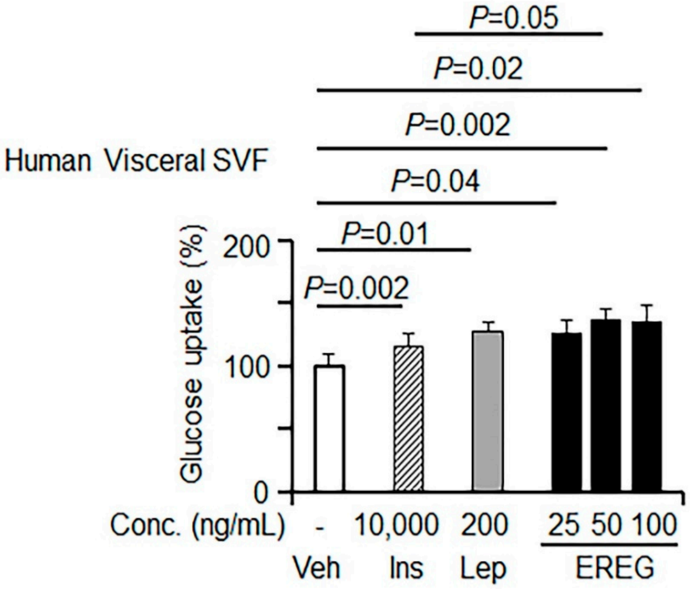

Supplemental Figure S5. EREG stimulated glucose uptake in human SVF cells isolated from visceral fat

Fluorescently-labelled (FD) glucose uptake was measured in human SVF cells. Cells were isolated from visceral (omental) fat from different donors (n = 7). Human stromal vascular fraction (SVF) cells were isolated from the visceral fat of obese subjects by using type 1 collagenase (17100017, Thermo Fisher Scientific) following a published method {Yasmeen, 2012 #127}. Isolated cells were cultured in Preadipocyte Growth Medium-2 Bullet Kit (Lonza, PT-8002, supplemented with PT-9502; Basel, Switzerland). The medium was changed every 3 days prior to measurement of glucose uptake. Preadipocytes were treated with vehicle, human insulin (Ins, 10μg/mL), leptin (Lep, 200ng/mL) and different EREG concentrations for 30 min. Data are shown as % of glucose uptake in non-stimulated cells (Veh, 100%); mean ± SEM, n = 7 per condition, *t*-test.

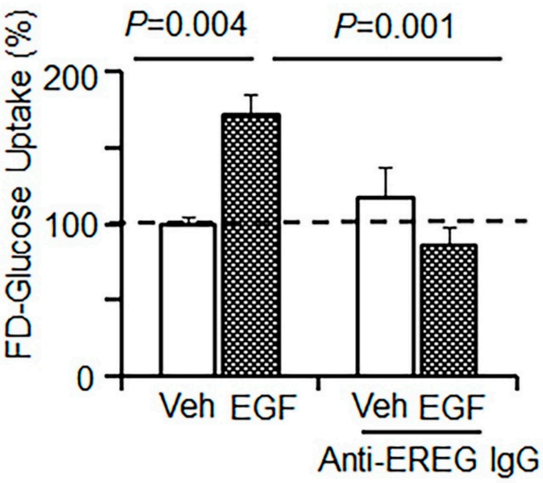

Supplemental Figure S6. EGF-mediated glucose uptake depends on EREG

FD-glucose uptake was measured in 3T3-L1 preadipocytes (n = 5/condition) with or without EGF (50 ng/mL) in the presence and absence of anti-EREG antibody (10μg/mL). Data (mean ± SD) are shown as a percent of control (Veh 100%). Unpaired Student's *t*-test.

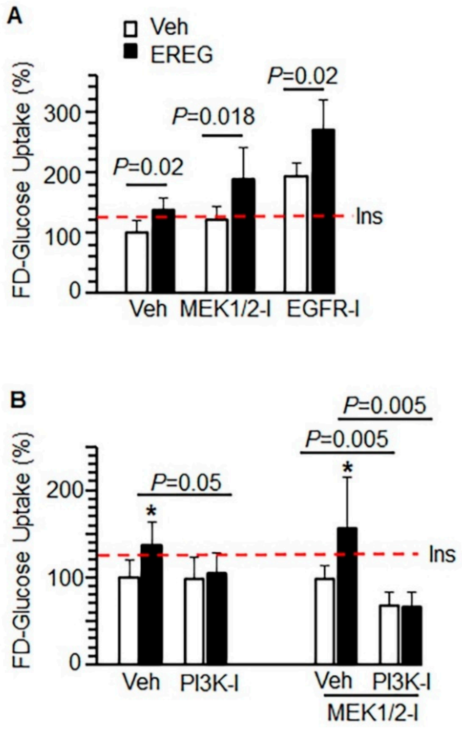

Supplemental Figure S7. EREG required PI3K but not EGFR and ERK1/2 for glucose uptake in human SVF cells

(**A**) Omental SVF preadipocytes from an obese and insulin resistant man were stimulated with and without EREG (50 ng/mL) in the presence and absence of inhibitors of MEK1/2 (U0126,10 μM) or EGFR (AG1478, 10 μM) for 30 min. FD-glucose uptake (n = 5) is shown as a percentaage of non-stimulated control (Veh, 100%, dashed line shows uptake in the presence of 10μg/mL insulin. Data are shown as a mean ± SEM. Independent *t*-test.

(**B**) Omental preadipocytes from an obese and insulin resistant woman were stimulated with and without EREG (50ng/mL) in the presence and absence of MAPK-I (U0126,10μM), and PI3K (wortmannin, 200nM) or their combination for 30min. FD-glucose uptake (n= 6) was calculated as a percentage of non-stimulated control (Veh,

100%, dashed line shows uptake in the presence of 10µg/mL insulin. Data are shown as mean ± SEM. Unpaired Student's *t*-test.

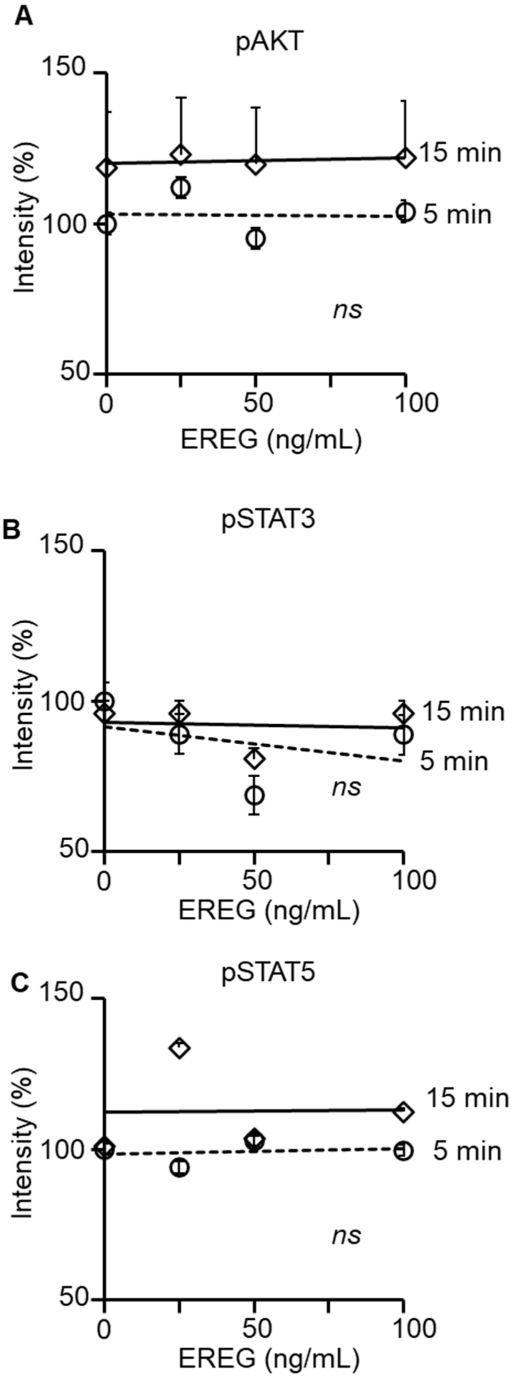

Supplemental Figure S8. The kinetics of pAKT (A), p-STAT3 (B), and p-STAT5 (C) was quantified based on the Western blots described in Figure 4C. Pearson correlation analysis, ns-not significant.

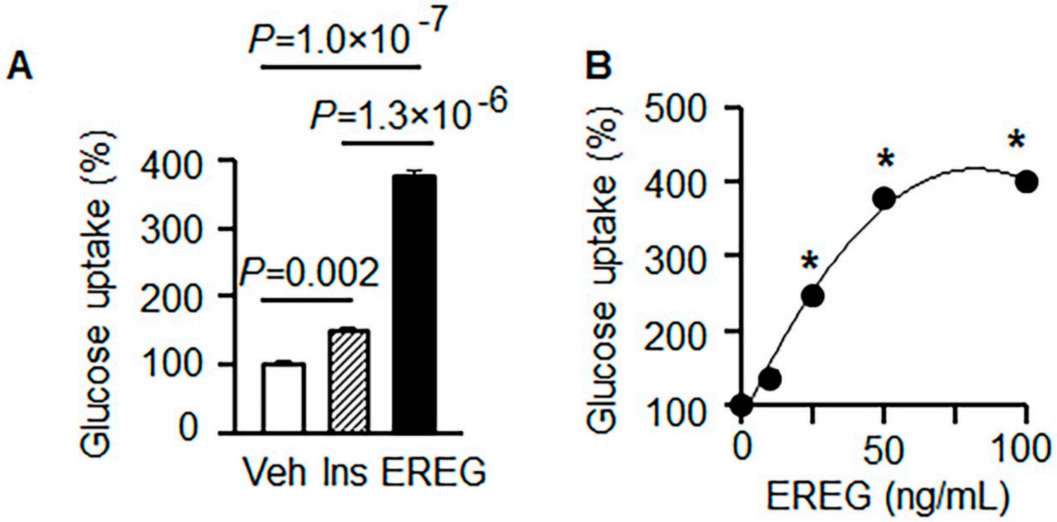

Supplemental Figure S9. EREG stimulated glucose uptake in mouse C2C12 muscle cells

(**A**) FD-glucose uptake by C2C12 cells after stimulation with insulin (Ins, 10μg/mL), EREG (50ng/mL) for 30 min. Data are shown as % of glucose uptake in non-stimulated cells (Veh, 100%); mean ± SEM, n = 7 per condition, independent *t*-test.

(**B**) Concentration-dependent increase in FD-glucose uptake by C2C12 cells stimulated with different concentrations of mouse EREG. Data are shown as a percentage of Veh-treated control (100%, mean, n = 6 per concentration). Asterisks represent significant differences compared to the vehicle group ($p < 0.05$, independent *t*-test).
